# Direct matching between the flow factor approach model and molecular dynamics simulation for nanochannel flows

**DOI:** 10.1038/s41598-021-04391-5

**Published:** 2022-01-10

**Authors:** Chuntao Jiang, Yongbin Zhang

**Affiliations:** 1 School of Mathematics and Statistics, Xinyang Normal University, Xinyang, Henan China; 2 College of Mechanical Engineering, Changzhou University, Changzhou, Jiangsu China

**Keywords:** Fluid dynamics, Computational nanotechnology

## Abstract

Mathematically formulating nanochannel flows is challenging. Here, the values of the characteristic parameters were extracted from molecular dynamics simulation (MDS), and directly input to the closed-form explicit flow factor approach model (FFAM) for nanochannel flows. By this way, the physical nature of the simulated system in FFAM is the same with that in MDS. Two nano slit channel heights respectively with two different liquid-channel wall interactions were addressed. The flow velocity profiles across the channel height respectively calculated from MDS and FFAM were compared. By introducing the equivalent value ${{\Delta_{im} } \mathord{\left/ {\vphantom {{\Delta_{im} } D}} \right. \kern-\nulldelimiterspace} D}$, FFAM fairly agrees with MDS for all the cases. The study values FFAM in simulating nanochannel flows.

## Introduction

Nanofluidics have been developed fast. They have the important applications in super purification, hemofiltration, DNA analysis, biosensors, drug delivery, heat and mass transfer, and micromachines etc.^1–8^. The studies on them have been mainly experimental or by molecular dynamics simulation (MDS)^1–15^. In these devices, the nanochannel flow has been shown by MDS to be distinctly different from the classical recognition^9–15^. It may be much slower or much faster than described by the classical hydrodynamic flow theory, depending on the fluid-channel coupling. The rheological properties including the viscosity and density of the confined fluid in a nanochannel are usually evolved due to the fluid-channel wall interaction; they are largely different from the fluid bulk values^16–18^. Also, the wall slippage easily occurs in a nanochannel^19,20^. It is believed that these new phenomena should be the important factors causing the very special nanochannel flow. It was also recognized that the effects of the discontinuity and inhomogeneity of the fluid across the channel height contribute very significantly to the nanochannel flow^21^. It was however felt a pity that no powerful constitutive models were available for efficiently analyzing the nanoscale flows in the realistic nanofluidics with the length on the micrometer or millimeter scales in one or two dimensions. This resulted in the lacking of the development of the systematic design theory for a lot of nanofluidic devices such as nanoporous filtration membranes and micro/nano bearings. The researches on these devices were mainly focused on the experimental manufacturing and tests but lacked intensive theoretical understanding^2–4,8^.

Molecular dynamics simulation has been popularly used in the study of nanochannel flows for capturing the local physical property evolution and the flow velocity profile of the confined fluid^9–15,22–35^. It can reveal the flow phenomena in a nanoscale zone; but it is hardly applicable for an engineering flow with macroscale sizes, because of the cost of both the over long computational time and the over large computer storage. It is mandatory to develop the feasible flow models for engineering nanochannel flows; they can be used in the theoretical research and in the design of relevant nanofluidics.

Zhang^36^ proposed the flow factor approach model (FFAM), an equivalently continuum model for nanochannel flows, for effectively analyzing the nanoscale flows in very small engineering surface separations. He considered both the discontinuity and inhomogeneity of the fluid across the channel height. He used the concept of the local viscosity, which is dependent on the local density; these two parameters both are dependent on the separation between the neighboring fluid molecules across the channel height. The coarse-graining or multiscale simulation methods were also proposed for saving the computational time and computer storage when simulating the nanochannel flows. Bhadauria and Aluru^37^ proposed a quasi-continuum hydrodynamic model for the nano slit pore flow; they computed the density profile and the viscosity change across the channel height; they also incorporated the wall slippage effect by using the Dirichlet type slip boundary condition. Ghorbanian et al.^38^ proposed a phenomenological continuum model for force-driven nanochannel flows; they used the effective channel height and the density deficit parameter based on the density layering near the channel walls; their model needs the calibration of the parameters by MDS. Kasiteropoulou et al.^39^ used the dissipative particle dynamics method to study the flow properties in a nanochannel. Swift et al.^40^ used the lattice Boltzman method to simulate the binary fluid system. Naris and Valougeorgis^41^ used the kinetic theory to simulate the non-equilibrium flow in a very narrow channel.

The present study focuses on FFAM for nanochannel flows. The previous studies^36,42–46^ have examined this model by comparing it with MDS by using the characteristic parameter values which were ever suspected by some readers to be fitting, although satisfactory agreements between these two approaches were existing. As a new corroboration work, the present paper presents the direct comparison between FFAM and MDS in the calculated flow velocity profiles, by directly inputting the values of the characteristic parameters extracted from MDS to FFAM. By this way, the averaged local density and local viscosity profiles across the channel height in FFAM are the same with those in MDS; in the comparison the physical structure of the simulated system in FFAM is forehand set as the same with that in MDS. The comparison shows that good agreements between these two approaches are existing. The present study not only directly substantiates FFAM but also confirms the earlier work^36,42–46^.

The importance of the present work is to provide the base stone for the theoretical derivation of the flow equation for the nanoscale flow which is based on FFAM, though this equation itself matched well with the MDS results^21,43^.

## The flow factor approach model for nanochannel flow

**Figure 1:**
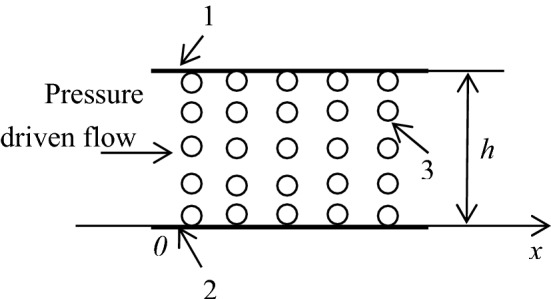
The flow factor approach model (FFAM) for the flow in a nano slit pore^36,42^. 1-upper plane surface, 2-lower plane surface, 3-ordered fluid molecule.

For a better understanding, FFAM is introduced in this section. This model has been shown in details in Refs.^21,36,42^. Here, it is only briefly repeated. Figure 1 shows the flow factor approach model for the flow in a nano slit pore, where the molecules of the fluid with the equivalent number $n$ across the channel height are ordered normal to the channel wall; because of the interaction between the fluid and the channel wall, the separation between the neighboring fluid molecules across the channel height is varied; this results in the density and viscosity variations across the channel height.

According to the model, in the pressure driven flow shown in Fig. 1, the velocity of the fluid molecule is^21,36,42^:

1 $$\begin{aligned} u_{i} &= \overline{u}_{b} + \frac{{i(\overline{u}_{a} - \overline{u}_{b} )(\Delta_{l} /\eta_{line,l} )_{avr,i} }}{{(n - 1)(\Delta_{l} /\eta_{line,l} )_{avr,n - 1} }} + Di\frac{\partial p}{{\partial x}}(l\Delta_{l - 1} /\eta_{line,l - 1} )_{avr,i} \\ &\quad \times \,\left[ {1 - \frac{{(\Delta_{l} /\eta_{line,l} )_{avr,i} (l\Delta_{l - 1} /\eta_{line,l - 1} )_{avr,n - 1} }}{{(\Delta_{l} /\eta_{line,l} )_{avr,n - 1} (l\Delta_{l - 1} /\eta_{line,l - 1} )_{avr,i} }}} \right]\quad {\text{for}}\,i = {1},{ 2}, \ldots , \, \left( {n - {1}} \right). \\ \end{aligned}$$

Here, $i$ is the order number of the fluid molecule across the channel height, $\overline{u}_{a}$ is the velocity of the (*n* − 1)th fluid molecule (on the upper solid wall), $\overline{u}_{b}$ is the velocity of the 0th fluid molecule (on the lower solid wall), $p$ is the driving pressure, $D$ is the fluid molecule diameter, $x$ is the coordinate shown in Fig. 1, and $\eta_{line,i - 1}$ and $\Delta_{i - 1}$ are respectively the local viscosity and the separation between the *i*th and (*i* − 1)th fluid molecules across the channel height. It is formulated that:

2 $$(\Delta_{l} /\eta_{line,l} )_{avr,i} = \frac{{\sum\limits_{l = 0}^{i - 1} {\Delta_{l} /\eta_{line,l} } }}{i},$$

and

3 $$(l\Delta_{l - 1} /\eta_{line,l - 1} )_{avr,i} = \frac{{\sum\limits_{l = 1}^{i} {l\Delta_{l - 1} /\eta_{line,l - 1} } }}{i}.$$

In this model, it is assumed that the distribution of the separation between the neighboring fluid molecules across the channel height is symmetrical with respect to the median plane of the channel; it is taken that $\Delta_{i + 1} /\Delta_{i} = q_{0} > 1$ and $\eta_{line,i} /\eta_{line,i + 1} = q_{0}^{m} > 1$ (for *i* = 0, 1,…, (*n* − 1)/2–2). Here, $q_{0}$ and *m* are respectively constant, and *n* is an odd number ($n \ge 5$). The values of $\Delta_{i + 1} /\Delta_{i}$ and $\eta_{line,i} /\eta_{line,i + 1}$ both may be varied across the channel height; the model takes $q_{0}$ as the average value of $\Delta_{i + 1} /\Delta_{i}$ (for *i* = 0, 1,…, (*n* − 1)/2–2).

As the flow in FFAM is essentially molecular-scale and non-continuum, the dimension of the channel height in FFAM should be no more than the total thickness of the physical adsorbed layers respectively freely formed on both the upper and lower channel wall surfaces. Equation (1) shows that for no wall slippage i.e. $\overline{u}_{a} = \overline{u}_{b} = 0$, once the values of *D*, *m*, *n*, $q_{0}$, $\Delta_{im} /D$, $\eta_{line,im}$ and $\partial p/\partial x$ are known, the flow velocity $u_{i}$ of each fluid molecule across the channel height can be directly calculated^36,42^. These input parameters are the characteristic parameters in FFAM. The values of *D*, *n* and $\partial p/\partial x$ can be determined beforehand. While, the values of *m*, $q_{0}$, $\Delta_{im} /D$ and $\eta_{line,im}$ can be calibrated by MDS or found/deduced from other scientific data sources. The natures of both the flowing liquid and the channel wall determine the values of all these characteristic parameters except $\partial p/\partial x$. By setting the values of these characteristic parameters in FFAM as the same with those in MDS, the physical nature of the simulated system in FFAM is the same with that in MDS.

## Molecular dynamics simulation

### Simulation method and calculated parameters

For comparison with FFAM, molecular dynamics simulation was made for the nano slit pore flow. This section presents the MDS details. Here, the methane nanochannel flow was simulated by the non-equilibrium MDS. The periodic boundary was implemented to the simulation system except for the confined methane molecules in the z-direction. The methane molecules were arranged as a face-centered cubic between two paralled silicon atom flats. The modification of the optimized potential for the liquid simulation (MOPLS) model^47^ was used in this work. It is written as:

4 $$U_{{{\text{MOPLS}}}} \left( r \right) = \sum\limits_{\alpha ,\beta } {\sum\limits_{i \in \alpha ,j \in \beta } {\left( {4\varepsilon_{ab} \left( {\left( {{{\sigma_{ab} } \mathord{\left/ {\vphantom {{\sigma_{ab} } {r_{ij} }}} \right. \kern-\nulldelimiterspace} {r_{ij} }}} \right)^{12} - \left( {{{\sigma_{ab} } \mathord{\left/ {\vphantom {{\sigma_{ab} } {r_{ij} }}} \right. \kern-\nulldelimiterspace} {r_{ij} }}} \right)^{6} } \right) + {{q_{i} q_{j} } \mathord{\left/ {\vphantom {{q_{i} q_{j} } {4\pi \varepsilon_{0} r_{ij} }}} \right. \kern-\nulldelimiterspace} {4\pi \varepsilon_{0} r_{ij} }}} \right)} } ,$$

where $\sigma$, $\varepsilon$, $\varepsilon_{0}$, $q$ and $r_{ij}$ are respectively the length unit, the depth of the interaction energy well, the permittivity of vacuum, point charge and the distance between the atoms $i$ and $j$. The subscripts $a$ and $b$ respectively represent C and H. $\alpha$ and $\beta$ denote the interaction between different methane molecules. Moreover, in the potential model, the bond length is $l_{{{\text{CH}}}} = 1.087\;{\AA}$, and ${\kern 1pt} q_{{\text{C}}} = - \,4q_{{\text{H}}} = - \,0.572\;e$ ($e = 4.803 \times 10^{ - 10} \;{\text{esu}}$). The intermolecular potential parameters are given in Table 1.

**Table 1:** MOPLS potential parameters^47^.

Type	Parameter	C–C	C–H	H–H
MOPLS	${\varepsilon \mathord{\left/ {\vphantom {\varepsilon {k_{B} }}} \right. \kern-\nulldelimiterspace} {k_{B} }}\left( {\text{K}} \right)$	46.8	17.17	6.30
	$\sigma \left( {\AA} \right)$	3.45	3.06	2.67

**Figure 2:**
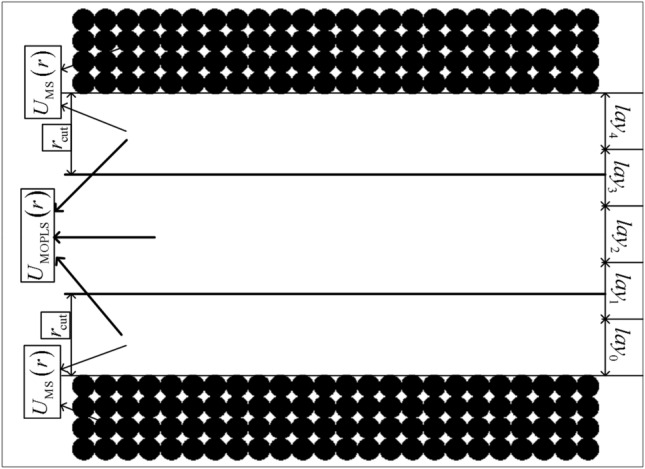
Schematic of the methane molecules confined by two parallel silicon atom flats.

Figure 2 shows the interactions among different particles when using the non-equilibrium MDS; it also shows the layers used for calculating the relevant parameters of FFAM. In order to solve the interaction problem between the wall atom and the methane molecule, the non-equilibrium multiscale MDS was applied in this work; the intermolecular force between the wall atom and the methane molecule was computed using the methane coarse-graining potential, coupled with the Lorentz- Berthelot mixing rule nearby the wall (see Fig. 1 in Ref.^48^). The interaction between the wall atom and the methane molecule was modeled by the following equation:

5 $$U_{{{\text{MS}}}} \left( r \right) = 4\chi \varepsilon_{{{\text{MS}}}} \left( {\left( {\frac{{\sigma_{{{\text{MS}}}} }}{r}} \right)^{12} - \left( {\frac{{\sigma_{{{\text{MS}}}} }}{r}} \right)^{6} } \right).$$

The parameters $\varepsilon_{{{\text{MS}}}}$ and $\sigma_{{{\text{MS}}}}$ were obtained by the Lorentz–Berthelot mixing rule:

6 $$\varepsilon_{{{\text{MS}}}} = \sqrt {\varepsilon_{{{\text{CG}}}} \cdot \varepsilon_{{\text{S}}} } ,\quad \,\sigma_{{{\text{MS}}}} = {{\left( {\sigma_{{{\text{CG}}}} + \sigma_{{\text{S}}} } \right)} \mathord{\left/ {\vphantom {{\left( {\sigma_{{{\text{CG}}}} + \sigma_{{\text{S}}} } \right)} 2}} \right. \kern-\nulldelimiterspace} 2},$$

where $\chi$ is the interaction strength factor between the wall atom and the methane molecule. In this study, $\chi = 1.0$ and $\chi = 2.0$ were respectively employed. The methane coarse-graining potential parameters $\varepsilon_{{{\text{CG}}}} = 1.329\;{{{\text{kJ}}} \mathord{\left/ {\vphantom {{{\text{kJ}}} {{\text{mol}}}}} \right. \kern-\nulldelimiterspace} {{\text{mol}}}}$ and $\sigma_{{{\text{CG}}}} = 3.645\;{\AA}$ were optimized, by using the relative entropy coarse-graining framework^49,50^ in the bulk methane ensemble at the initial condition $\rho = 377.15\;{\text{kg/m}}^{3}$ and $T = 140\;{\text{K}}$. The details of the methane coarse-graining process have been shown in Ref.^49^. The silicon atom intermolecular potential parameters are: $\varepsilon_{{\text{S}}} = 1.6885\;{\text{kJ/mol}}$ and $\sigma_{{\text{S}}} = 3.826\;{\AA}$^51^. An ABAB stacking configuration was used to describe the silicon atom flat walls; moreover, every atom was conducted by the harmonic potential site ${\mathbf{r}}_{{{\text{eq}}}}$ as follows:

7 $$u\left( {\left| {{\mathbf{r}}\left( t \right) - {\mathbf{r}}_{{{\text{eq}}}} } \right|} \right) = \frac{1}{2}k_{{\text{w}}} \left( {\left| {{\mathbf{r}}\left( t \right) - {\mathbf{r}}_{{{\text{eq}}}} } \right|} \right)^{2} .$$

Here, ${\mathbf{r}}\left( t \right)$ is the atom position at time $t$, and $k_{{\text{w}}} = 72\varepsilon_{{\text{S}}} /\left( {2^{{{1 \mathord{\left/ {\vphantom {1 3}} \right. \kern-\nulldelimiterspace} 3}}} \sigma_{{\text{S}}}^{2} } \right)$ was obtained by using the second derivative value of the silicon atom intermolecular potential at $r = r_{0} = 2^{{{1 \mathord{\left/ {\vphantom {1 6}} \right. \kern-\nulldelimiterspace} 6}}} \sigma_{{\text{S}}}$. To avoid the effect of the compressibility of the methane flowing through the nanochannel, the external driving body force $f_{{\text{x}}} = 0.075\;\left( {{\varepsilon \mathord{\left/ {\vphantom {\varepsilon \sigma }} \right. \kern-\nulldelimiterspace} \sigma }} \right)$ was employed in the *x* direction^52^. The quaternion parameters were applied to calculate the orientation coordinates for the methane (tetrahedral) molecule. The fourth Predictor Corrector method was used for solving the motion equations of the methane molecule. The temperature of the simulation system was controlled by the NVT ensemble with a Nosé–Hoover thermostat. In order to check the simulation system temperature, the thermal kinetic energies per molecule were compared in the *y* and *z* directions; they are respectively ${{mv_{y}^{2} } \mathord{\left/ {\vphantom {{mv_{y}^{2} } 2}} \right. \kern-\nulldelimiterspace} 2} = {{k_{B} T} \mathord{\left/ {\vphantom {{k_{B} T} 2}} \right. \kern-\nulldelimiterspace} 2}$ and ${{mv_{z}^{2} } \mathord{\left/ {\vphantom {{mv_{z}^{2} } 2}} \right. \kern-\nulldelimiterspace} 2} = {{k_{B} T} \mathord{\left/ {\vphantom {{k_{B} T} 2}} \right. \kern-\nulldelimiterspace} 2}$^53,54^ ($m$ is the mass of methane molecule, $T$ is the system temperature, and $v_{y}$ and $v_{z}$ are the velocities in the *y* and *z* directions respectively). In this simulation, the reduced units are $\sigma = 4.01{\kern 1pt} \;{\AA}$ and ${\varepsilon \mathord{\left/ {\vphantom {\varepsilon {k_{B} }}} \right. \kern-\nulldelimiterspace} {k_{B} }} = 142.87\;{\text{K}}$; The simulation step is $\Delta t = 0.001$ ($1.3 \times 10^{ - 15} \;{\text{s}}$), and the energy cutoff circle radius is $r_{{{\text{cut}}}} = 2.5\;\sigma$. In order to obtain the accurate results statistically, the results for the initial $1.0 \times 10^{6}$ simulation steps were discarded; the relevant molecule information and parameters for FFAM were calculated in the next $5.0 \times 10^{5}$ simulation steps. In order to calculate the local viscosity for FFAM, the velocity profiles of the methane fluid were collected by dividing the distance between the two silicon atom flats into $560$ bins. Moreover, the nanochannel dimensions are $12 \times 12 \times 6$ and $12 \times 12 \times 7$ (the unit is $\sigma$) respectively, i.e. the distance between the two silicon atom flats is six or seven times of the methane molecule diameter. To obtain the relevant parameters $q_{0}$, $\eta_{line,j}$, $\Delta_{im} /D$ and $\eta_{line,im}$ of FFAM, we supposed the methane fluid layer is five ($n = 5$) when the system arrived at the equilibrium state for two cases. These parameters were respectively calculated by the following equations:

8 $$q_{0} = \frac{{\sum\nolimits_{l = 0}^{{\left( {\left( {n - 1} \right)/2} \right) - 2}} {\frac{{\Delta_{l + 1} }}{{\Delta_{l} }}} }}{{\frac{n - 1}{2} - 1}},$$

9 $$\Delta_{l} = \left\langle {\frac{1}{{n_{{lay,\;l{ + }1}} }}\frac{1}{{n_{lay,\;l} }}\sum\limits_{i = 0}^{{n_{{lay,\;l{ + }1}} }} {\sum\limits_{j = 0}^{{n_{lay,\;l} }} {\left( {{\mathbf{r}}_{l - 1,iz} - {\mathbf{r}}_{l,jz} } \right)} } } \right\rangle - D,$$

10 $${{\Delta_{im} } \mathord{\left/ {\vphantom {{\Delta_{im} } D}} \right. \kern-\nulldelimiterspace} D} = \frac{1}{2D}\left( {\Delta_{{\left( {n - 1} \right)/2}} + \Delta_{{\left( {\left( {n - 1} \right)/2 - 1} \right)}} } \right).$$

Here, $\Delta_{l}$ is the averaged gap between the ($l - 1$)th fluid layer and the *l*th fluid layer, the subscripts *z* indicates the position of the fluid molecule in the *z* direction, $D = 0.414\;{\text{nm}}$ (the methane molecule diameter), and 〈 〉 denotes the ensemble average. The local viscosities of the methane fluid were calculated by the Poiseuille flow method as follows^55^:

11 $$\eta _{{line,j}} = - {{\rho f_{x} } \mathord{\left/ {\vphantom {{\rho f_{x} } {2k_{{line,j}} }}} \right. \kern-\nulldelimiterspace} {2k_{{line,j}} }},$$

12 $$k_{line,j} = {{\left( {v_{x}^{{_{line,j} }} - v_{{0}}^{{_{line,j} }} } \right)} \mathord{\left/ {\vphantom {{\left( {v_{x}^{{_{line,j} }} - v_{{0}}^{{_{line,j} }} } \right)} {\left( {z_{line,j} - z_{o} } \right)}}} \right. \kern-\nulldelimiterspace} {\left( {z_{line,j} - z_{o} } \right)}}^{2}.$$

### Values of the characteristic parameters in FFAM extracted from MDS

For the direct comparison of FFAM with MDS, the input values of the characteristic parameters in FFAM must come from MDS so that these two models are convincing to be initially completely matching. Tables 2, 3, 4 and 5 respectively show the dimensionless separations between the neighboring fluid molecules across the channel height calculated from MDS; the calculated corresponding values of $q_{0}$ are respectively put below these tables. Tables 6, 7, 8 and 9 respectively show the values of the local viscosity ratio ${{\eta_{line,j} } \mathord{\left/ {\vphantom {{\eta_{line,j} } {\eta_{line,j + 1} }}} \right. \kern-\nulldelimiterspace} {\eta_{line,j + 1} }}$ calculated from MDS indicating the significant variation of the local viscosity across the channel height owing to the fluid-channel wall interaction^58^; the calculated corresponding values of $m$ are respectively put below these tables.

**Table 2:** The separations between the neighboring fluid molecules across the channel height calculated from MDS, $h$ = 2.484 nm, $\chi = 1.0$, $n$ = 5.

Separation	${{\Delta_{0} } \mathord{\left/ {\vphantom {{\Delta_{0} } D}} \right. \kern-\nulldelimiterspace} D}$	${{\Delta_{1} } \mathord{\left/ {\vphantom {{\Delta_{1} } D}} \right. \kern-\nulldelimiterspace} D}$	${{\Delta_{2} } \mathord{\left/ {\vphantom {{\Delta_{2} } D}} \right. \kern-\nulldelimiterspace} D}$	${{\Delta_{3} } \mathord{\left/ {\vphantom {{\Delta_{3} } D}} \right. \kern-\nulldelimiterspace} D}$
Value	0.43117	0.51157	0.51193	0.43167

$$q_{0}$$ = 1.1862.

**Table 3:** The separations between the neighboring fluid molecules across the channel height calculated from MDS, $h$ = 2.484 nm, $\chi = 2.0$, $n$ = 5.

Separation	${{\Delta_{0} } \mathord{\left/ {\vphantom {{\Delta_{0} } D}} \right. \kern-\nulldelimiterspace} D}$	${{\Delta_{1} } \mathord{\left/ {\vphantom {{\Delta_{1} } D}} \right. \kern-\nulldelimiterspace} D}$	${{\Delta_{2} } \mathord{\left/ {\vphantom {{\Delta_{2} } D}} \right. \kern-\nulldelimiterspace} D}$	${{\Delta_{3} } \mathord{\left/ {\vphantom {{\Delta_{3} } D}} \right. \kern-\nulldelimiterspace} D}$
Value	0.46511	0.52826	0.52809	0.46512

$$q_{0}$$ = 1.1356.

**Table 4:** The separations between the neighboring fluid molecules across the channel height calculated from MDS, $h$ = 2.898 nm, $\chi = 1.0$, $n$ = 5.

Separation	${{\Delta_{0} } \mathord{\left/ {\vphantom {{\Delta_{0} } D}} \right. \kern-\nulldelimiterspace} D}$	${{\Delta_{1} } \mathord{\left/ {\vphantom {{\Delta_{1} } D}} \right. \kern-\nulldelimiterspace} D}$	${{\Delta_{2} } \mathord{\left/ {\vphantom {{\Delta_{2} } D}} \right. \kern-\nulldelimiterspace} D}$	${{\Delta_{3} } \mathord{\left/ {\vphantom {{\Delta_{3} } D}} \right. \kern-\nulldelimiterspace} D}$
Value	0.58531	0.70889	0.70838	0.58549

$$q_{0}$$ = 1.2105.

**Table 5:** The separations between the neighboring fluid molecules across the channel height calculated from MDS, $h$ = 2.898 nm, $\chi = 2.0$, $n$ = 5.

Separation	${{\Delta_{0} } \mathord{\left/ {\vphantom {{\Delta_{0} } D}} \right. \kern-\nulldelimiterspace} D}$	${{\Delta_{1} } \mathord{\left/ {\vphantom {{\Delta_{1} } D}} \right. \kern-\nulldelimiterspace} D}$	${{\Delta_{2} } \mathord{\left/ {\vphantom {{\Delta_{2} } D}} \right. \kern-\nulldelimiterspace} D}$	${{\Delta_{3} } \mathord{\left/ {\vphantom {{\Delta_{3} } D}} \right. \kern-\nulldelimiterspace} D}$
Value	0.60681	0.72269	0.72285	0.6066

$$q_{0}$$ = 1.1913.

**Table 6:** The values of ${{\eta_{line,j} } \mathord{\left/ {\vphantom {{\eta_{line,j} } {\eta_{line,j + 1} }}} \right. \kern-\nulldelimiterspace} {\eta_{line,j + 1} }}$ calculated from MDS, $h$ = 2.484 nm, $\chi = 1.0$, $n$ = 5.

Ratio of local viscosity	${{\eta_{line,0} } \mathord{\left/ {\vphantom {{\eta_{line,0} } {\eta_{line,1} }}} \right. \kern-\nulldelimiterspace} {\eta_{line,1} }}$	${{\eta_{line,2} } \mathord{\left/ {\vphantom {{\eta_{line,2} } {\eta_{line,3} }}} \right. \kern-\nulldelimiterspace} {\eta_{line,3} }}$
Value	1.3913	0.7182 (= 1/1.3924)

Average value of the local viscosity ratio = 1.3918, *m* = 1.9362.

**Table 7:** The values of ${{\eta_{line,j} } \mathord{\left/ {\vphantom {{\eta_{line,j} } {\eta_{line,j + 1} }}} \right. \kern-\nulldelimiterspace} {\eta_{line,j + 1} }}$ calculated from MDS, $h$ = 2.484 nm, $\chi = 2.0$, $n$ = 5.

Ratio of local viscosity	${{\eta_{line,0} } \mathord{\left/ {\vphantom {{\eta_{line,0} } {\eta_{line,1} }}} \right. \kern-\nulldelimiterspace} {\eta_{line,1} }}$	${{\eta_{line,2} } \mathord{\left/ {\vphantom {{\eta_{line,2} } {\eta_{line,3} }}} \right. \kern-\nulldelimiterspace} {\eta_{line,3} }}$
Value	1.6248	0.6154 (= 1/1.625)

Average value of the local viscosity ratio = 1.6249, *m* = 3.818.

**Table 8:** The values of ${{\eta_{line,j} } \mathord{\left/ {\vphantom {{\eta_{line,j} } {\eta_{line,j + 1} }}} \right. \kern-\nulldelimiterspace} {\eta_{line,j + 1} }}$ calculated from MDS, $h$ = 2.898 nm, $\chi = 1.0$, $n$ = 5.

Ratio of local viscosity	${{\eta_{line,0} } \mathord{\left/ {\vphantom {{\eta_{line,0} } {\eta_{line,1} }}} \right. \kern-\nulldelimiterspace} {\eta_{line,1} }}$	${{\eta_{line,2} } \mathord{\left/ {\vphantom {{\eta_{line,2} } {\eta_{line,3} }}} \right. \kern-\nulldelimiterspace} {\eta_{line,3} }}$
Value	1.3011	0.7624 (= 1/1.3116)

Average value of the local viscosity ratio = 1.3064, *m* = 1.3992.

**Table 9:** The values of $\eta_{line,j} /\eta_{line,j + 1}$ calculated from MDS, $h$ = 2.898 nm, $\chi = 2.0$, $n$ = 5.

Ratio of local viscosity	${{\eta_{line,0} } \mathord{\left/ {\vphantom {{\eta_{line,0} } {\eta_{line,1} }}} \right. \kern-\nulldelimiterspace} {\eta_{line,1} }}$	${{\eta_{line,2} } \mathord{\left/ {\vphantom {{\eta_{line,2} } {\eta_{line,3} }}} \right. \kern-\nulldelimiterspace} {\eta_{line,3} }}$
Value	1.7175	0.5822 (= 1/1.7176)

Average value of the local viscosity ratio = 1.7176, *m* = 3.09.

The values of $\Delta_{im} /D$ (*im* = (*n* − 1)/2) calculated from MDS are respectively:

- for $h$ = 2.484 nm and $\chi = 1.0$, $\frac{{\Delta_{im} }}{D} = { 0}{\text{.5118}}$,
- for $h$ = 2.484 nm and $\chi = 2.0$, $\frac{{\Delta_{im} }}{D} = { 0}{\text{.5282}}$,
- for $h$ = 2.898 nm and $\chi = 1.0$, $\frac{{\Delta_{im} }}{D} = { 0}{\text{.7086}}$,
- for $h$ = 2.898 nm and $\chi = 2.0$, $\frac{{\Delta_{im} }}{D} = { 0}{\text{.7228}}$.

The values of $\eta_{line,im}$ calculated from MDS are respectively:

- for $h$ = 2.484 nm and $\chi = 1.0$, $\eta_{line,im} = 7.6863E - 5\,{\text{Pa}} \cdot {\text{s}}$,
- for $h$ = 2.484 nm and $\chi = 2.0$, $\eta_{line,im} = 7.9162E - 5\,{\text{Pa}} \cdot {\text{s}}$,
- for $h$ = 2.898 nm and $\chi = 1.0$, $\eta_{line,im} = 7.3257E - 5\,{\text{Pa}} \cdot {\text{s}}$,
- for $h$ = 2.898 nm and $\chi = 2.0$, $\eta_{line,im} = 7.7133E - 5\,{\text{Pa}} \cdot {\text{s}}$.

In the present study, the above values of *m*, $q_{0}$, $\Delta_{im} /D$ and $\eta_{line,im}$ calculated by MDS are directly put into Eq. (1) (FFAM); also the values of *D*, *n* and $\partial p/\partial x$ in FFAM are chosen as the same with those in MDS. By this way, as FFAM describes, the averaged local density and local viscosity profiles across the channel height in FFAM are the same with those in MDS, and the physical nature of the simulated system in FFAM is the same with that in MDS. These are sufficient as the flow velocity profile across the channel height is determined by the averaged density profile in the median section of the channel height, not by the density profile near the solid walls which might be highly oscillatory^59^.

## Comparison of FFAM with MDS

The values of the characteristic parameters in FFAM shown above were input to FFAM^21,36,42^ to calculate the flow velocities of the fluid molecules across the channel height. These calculated flow velocities were compared with those directly calculated from MDS for the same case. In FFAM, it was taken that $n$ = 5, and the pressure gradient for the Poiseuille flow was taken exactly the same as in MDS i.e. $\partial p/\partial x = - \,3.10621E + 15\,{\text{Pa}} / {\text{m}}$.

### For the case $h$ = 2.484 nm

**Figure 3:**
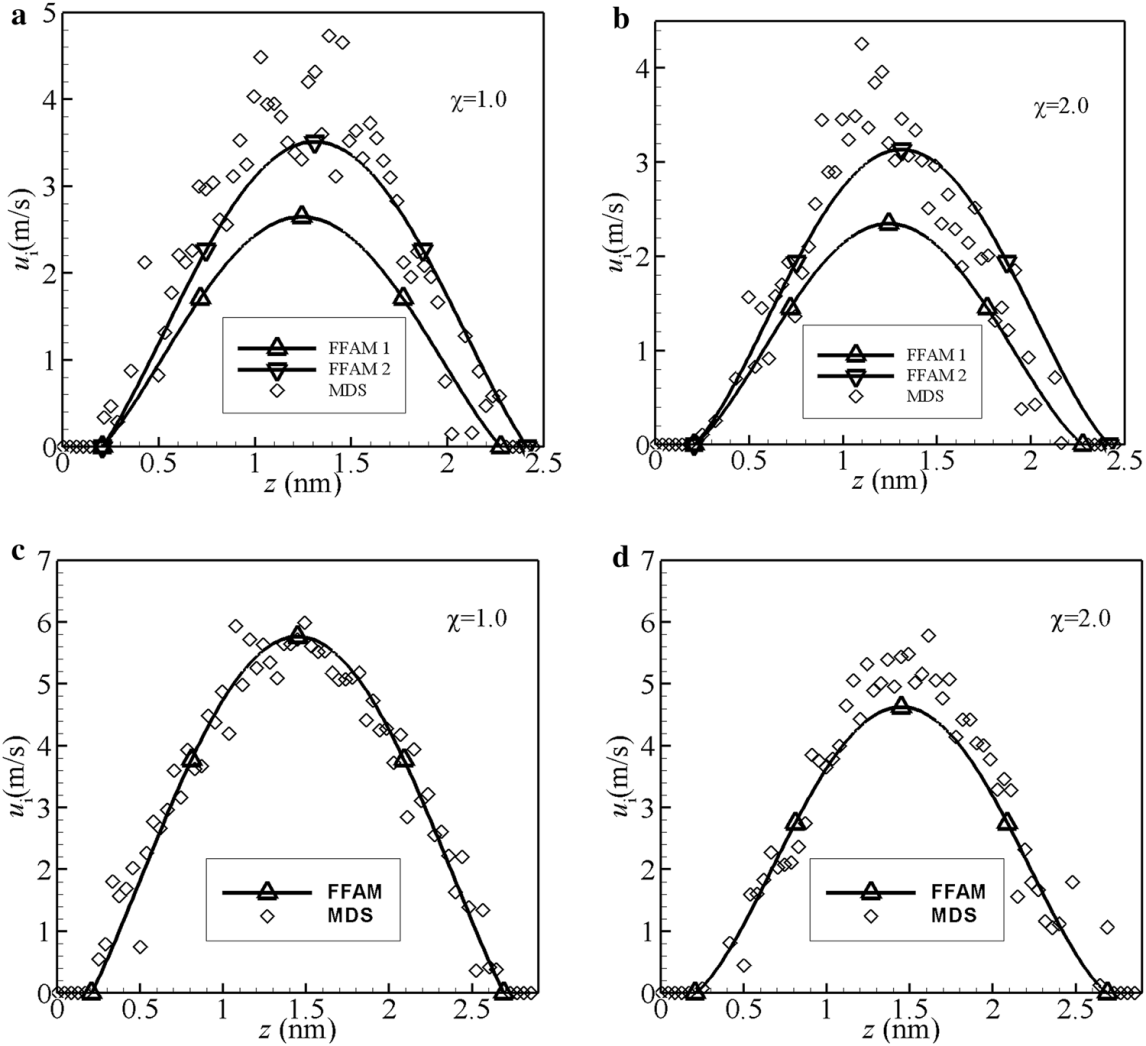
Comparisons between the flow factor approach model (FFAM) and molecular dynamics simulation (MDS) in the flow velocity profiles across the channel height. (**a**) $h$ = 2.484 nm; FFAM 1: ${{\Delta_{im} } \mathord{\left/ {\vphantom {{\Delta_{im} } D}} \right. \kern-\nulldelimiterspace} D} = 0.2713$, FFAM 2: ${{\Delta_{im} } \mathord{\left/ {\vphantom {{\Delta_{im} } D}} \right. \kern-\nulldelimiterspace} D} = 0.36$; (**b**) $h$ = 2.484 nm; FFAM 1: ${{\Delta_{im} } \mathord{\left/ {\vphantom {{\Delta_{im} } D}} \right. \kern-\nulldelimiterspace} D} = 0.2659$, FFAM 2: ${{\Delta_{im} } \mathord{\left/ {\vphantom {{\Delta_{im} } D}} \right. \kern-\nulldelimiterspace} D} = 0.355$; (**c**) $h$ = 2.898 nm; (**d**) $h$ = 2.898 nm.

Figure 3a shows the comparison between the flow velocities respectively calculated from FFAM and MDS for the case $h$ = 2.484 nm and $\chi = 1.0$. In the figure, the legend “FFAM 1” refers to the calculation from FFAM by using ${{\Delta_{im} } \mathord{\left/ {\vphantom {{\Delta_{im} } D}} \right. \kern-\nulldelimiterspace} D} = 0.2713$. For this case, the value of ${{\Delta_{im} } \mathord{\left/ {\vphantom {{\Delta_{im} } D}} \right. \kern-\nulldelimiterspace} D}$ calculated from MDS is actually 0.5118 as shown above; the reason for using the corrected value ${{\Delta_{im} } \mathord{\left/ {\vphantom {{\Delta_{im} } D}} \right. \kern-\nulldelimiterspace} D} = 0.2713$ in FFAM is to satisfy the channel height 2.484 nm, because ${{\Delta_{im} } \mathord{\left/ {\vphantom {{\Delta_{im} } D}} \right. \kern-\nulldelimiterspace} D} = 0.5118$ gives the channel height significantly greater than 2.484 nm in FFAM. The reason for this correction is that in actual cases, not completely like as assumed in FFAM, the fluid molecules are often not exactly ordered normal to the channel wall surface. In FFAM, if we use ${{\Delta_{im} } \mathord{\left/ {\vphantom {{\Delta_{im} } D}} \right. \kern-\nulldelimiterspace} D} = 0.5118$, for giving the channel height 2.484 nm, the equivalent number of the fluid molecules across the channel height should be $n_{eq}$ = 4.11; if we use *n* = 5, the value of ${{\Delta_{im} } \mathord{\left/ {\vphantom {{\Delta_{im} } D}} \right. \kern-\nulldelimiterspace} D}$ must thus be corrected for giving the channel height 2.484 nm. Figure 3a shows that the velocities calculated by “FFAM 1” are considerably smaller than those calculated from MDS especially in the central region of the channel. We checked that the reason for this discrepancy is that the ${{\Delta_{im} } \mathord{\left/ {\vphantom {{\Delta_{im} } D}} \right. \kern-\nulldelimiterspace} D}$ value 0.2713 in FFAM 1 is over lower than that (0.5118) calculated from MDS. Thus for this case, in FFAM, it is not rational to use ${{\Delta_{im} } \mathord{\left/ {\vphantom {{\Delta_{im} } D}} \right. \kern-\nulldelimiterspace} D} = 0.2713$ to calculate the flow velocities. In Fig. 3a, we also present the calculation results from FFAM by using ${{\Delta_{im} } \mathord{\left/ {\vphantom {{\Delta_{im} } D}} \right. \kern-\nulldelimiterspace} D} = 0.36$, as denoted by the legend “FFAM 2”. It is shown that the results calculated by “FFAM 2” fairly agree with those calculated from MDS. The reason for this much better matching should be the closer value of ${{\Delta_{im} } \mathord{\left/ {\vphantom {{\Delta_{im} } D}} \right. \kern-\nulldelimiterspace} D}$ used in “FFAM 2” than in “FFAM 1”. We examined that in “FFAM 2” the channel height is increased only by 8% as compared to the exact value 2.484 nm.

The same circumstances also exist in Fig. 3b, which is for the case $h$ = 2.484 nm and $\chi = 2.0$. In Fig. 3b, the results calculated by “FFAM 1” are considerably smaller than those calculated from MDS, due to the used ${{\Delta_{im} } \mathord{\left/ {\vphantom {{\Delta_{im} } D}} \right. \kern-\nulldelimiterspace} D}$ value 0.2659 over lower than that (0.5282) calculated from MDS; while, the results calculated by “FFAM 2” fairly agree with those calculated from MDS, due to the used greater but closer ${{\Delta_{im} } \mathord{\left/ {\vphantom {{\Delta_{im} } D}} \right. \kern-\nulldelimiterspace} D}$ value 0.355. It was checked that for this case, in “FFAM 2”, the channel height is increased only by 8%.

### For the case $h$ = 2.898 nm

Figure 3c shows the comparison between FFAM and MDS for the case $h$ = 2.898 nm and $\chi = 1.0$. It is shown that FFAM fairly agrees with MDS. In FFAM, the value of ${{\Delta_{im} } \mathord{\left/ {\vphantom {{\Delta_{im} } D}} \right. \kern-\nulldelimiterspace} D}$ is 0.5476 for giving the channel height 2.898 nm; this ${{\Delta_{im} } \mathord{\left/ {\vphantom {{\Delta_{im} } D}} \right. \kern-\nulldelimiterspace} D}$ value is relatively close to that (0.7086) calculated from MDS.

Figure 3d also shows the good matching between FFAM and MDS for the case $h$ = 2.898 nm and $\chi = 2.0$. In FFAM, the value of ${{\Delta_{im} } \mathord{\left/ {\vphantom {{\Delta_{im} } D}} \right. \kern-\nulldelimiterspace} D}$ is 0.5436 for giving the channel height; it is relatively close to the ${{\Delta_{im} } \mathord{\left/ {\vphantom {{\Delta_{im} } D}} \right. \kern-\nulldelimiterspace} D}$ value (0.7228) calculated from MDS.

From Fig. 3a–d, it can be found that with the increase of the fluid-channel wall interaction strength i.e. with the increase of $\chi$ the flow velocities across the channel height are significantly reduced; this shows the varying transport properties of the confined fluid influenced by the fluid-channel wall interaction^58^. Such a flow characteristic is actually reflected by the values of *m*, $q_{0}$, $\Delta_{im} /D$ and $\eta_{line,im}$ in FFAM as shown above. Different hydrophilicity or hydrophobicity of the channel wall, different values of *m*, $q_{0}$, $\Delta_{im} /D$ and $\eta_{line,im}$ in FFAM; if the wall slippage occurs, $\overline{u}_{a}$ and/or $\overline{u}_{b}$ are not vanishing and they are the wall slipping velocities required to be solved based on the limiting interfacial shear strength model according to FFAM^60^.

## Commenting remarks

The above comparisons directly validate FFAM. The subsequently made theoretical derivations^21,43^ of the flow equation for the nanoscale flow based on FFAM thus firmly stand. The virtues of FFAM is the flow equation it gives^21,43^, which is the governing equation for the nanoscale flow and for the two-dimensional flow reads:

13 $$q_{m} = \frac{{\overline{u}_{a} + \overline{u}_{b} }}{2}h\rho_{bf}^{eff} + \frac{{S\rho_{bf}^{eff} h^{3} }}{{12\eta_{bf}^{eff} }}\frac{dp}{{dx}},$$

where $q_{m}$ is the mass flow rate per unit channel length, $\overline{u}_{a}$ and $\overline{u}_{b}$ are respectively the velocities of the fluid molecules on the upper and lower channel walls, $h$ is the channel height, $dp/dx$ is the driving pressure gradient ($p$ is the pressure and $x$ is the coordinate), $\rho_{bf}^{eff}$ and $\eta_{bf}^{eff}$ are respectively the average density and the effective viscosity of the fluid across the channel height, and $S$ is the parameter characterizing the non-continuum effect across the channel height.

In the application of FFAM^56,57^, we normally do not need to obtain the input parameter values calibrated from MDS as shown in the above section. Mostly by other way, as shown in Eq. (13), $\rho_{bf}^{eff}$ and $\eta_{bf}^{eff}$ can respectively be formulated as the functions of the channel height $h$ by the regression equations based on the experimentally measured, MDS or FFAM calculated values of them, $S$ can also be regressed out as the function of $h$ based on the MDS or FFAM calculation results, and $\overline{u}_{a}$ and $\overline{u}_{b}$ can respectively be solved based on the no-slip or slip boundary conditions. When the formulations for all these parameters are given, Eq. (13) can be applied for solving the nanoscale flow problem in engineering such as in nanoporous filtration membranes, cellular connexon and membranes and micro/nano bearings etc., which are not solvable by MDS due to the limitation by the computational capacity. The advantage of Eq. (13) (or FFAM) is its convenience for calculating the nanoscale flow problem, just like we solve the flow problem by using the classical continuum hydrodynamic equation. These are obviously substantially progressive towards solving the engineering nanoscale flow problems, not affordable by MDS.

## Conclusions

The flow velocities in the nano slit pores in the Poiseuille flow were calculated from the flow factor approach model (FFAM) by using all the input parameter values obtained from molecular dynamics simulation (MDS). As a new progressive comparison work, in the present study the physical nature of the simulated system including the averaged local density and local viscosity profiles across the channel height in FFAM was forehand set as the same with that in MDS. It was found that FFAM well agrees with MDS because of the very close flow velocities calculated from these two approaches for the same cases. Since FFAM is the equivalent model for a nanochannel flow and the fluid molecules across the channel height in an actual case is often not exactly ordered normal to the channel wall surface, in FFAM the value of ${{\Delta_{im} } \mathord{\left/ {\vphantom {{\Delta_{im} } D}} \right. \kern-\nulldelimiterspace} D}$ should often be required to be corrected to give the correct channel height; it can be a little smaller than that calculated from MDS; this ${{\Delta_{im} } \mathord{\left/ {\vphantom {{\Delta_{im} } D}} \right. \kern-\nulldelimiterspace} D}$ value should be understood as the equivalent value.

As a substantiated model, FFAM can be successfully used in the modeling of nanoscale flows such as occurring in nanoporous filtration membranes^56^, cellular connexons and membranes and micro/nano bearings^57^ etc. As a constitutive model, FFAM radically alters the method of the modeling of the molecular scale flow compared to MDS. The advantage of FFAM is its high efficiency in the engineering nanoscale flow modeling just with the modest cost of computational time and computer storage^56,57^, not owned by MDS^9–15^. FFAM should become a reliable analytical tool for studying the relevant subjects in nanofluidics.

## References

[1] Baker LA, Bird SP (2008). Nanopores: A makeover for membranes. Nat. Nanotechnol..

[2] Jackson EA, Hillmyer MA (2010). Nanoporous membranes derived from block copolymers: From drug delivery to water filtration. ACS Nano.

[3] Fissel WH, Dubnisheva A, Eldridge AN, Fleischman AJ, Zydney AL, Roy S (2009). High-performance silicon nanopore hemofiltration membranes. J. Membr. Sci..

[4] Yang SY, Ryu I, Kim HY, Kim JK, Jang SK, Russell TP (2006). Nanoporous membranes with ultrahigh selectivity and flux for the filtration of viruses. Adv. Mater..

[5] Tegenfeldt J, Prinz C, Cao H, Huang RL, Austin RH, Chou SY, Cox EC, Sturm JC (2004). Micro- and nanofluidics for DNA analysis. Anal. Bioanal. Chem..

[6] Yanik AA, Huang M, Artar A, Chang TY, Altug H (2010). Integrated nanoplasmonic-nanofluidic biosensors with targeted delivery of analytes. Appl. Phys. Lett..

[7] Morikawa K, Kazoe Y, Takagi Y, Tsuyama Y, Pihosh Y, Tsukahara T, Kitamori T (2020). Advanced top-down fabrication for a fused silica nanofluidic device. Micromachines.

[8] Cadotte JE, Petersen RJ, Larson RE, Erickson EE (1980). A new thin film composite seawater reverse osmosis membrane. Desalination.

[9] Bitsanis I, Magda JJ, Tirrell M, Davis HT (1987). Molecular dynamics of flow in micropores. J. Chem. Phys..

[10] Bitsanis I, Vanderlick TK, Tirrell M, Davis HT (1988). A tractable molecular theory of flow in strongly inhomogeneous fluids. J. Chem. Phys..

[11] Somers SA, Davis HT (1992). Microscopic dynamics of fluids confined between smooth and atomically structured solid surfaces. J. Chem. Phys..

[12] Takaba H, Onumata Y, Nakao S (2007). Molecular simulation of pressure-driven fluid flow in nanoporous membranes. J. Chem. Phys..

[13] Whitby M, Quirke N (2007). Fluid flow in carbon nanotubes and nanopipes. Nat. Nanotechnol..

[14] Mattia D, Calabro F (2012). Explaining high flow rate of water in carbon nanotubes via solid–liquid molecular interactions. Microfluid. Nanofluid..

[15] Mattia D, Lee KP, Calabro F (2014). Water permeation in carbon nanotube membranes. Curr. Opin. Chem. Eng..

[16] Jabbarzadeh A, Atkinson JD, Tanner RI (1997). Rheological properties of thin liquid films by molecular dynamics simulations. J. Non-Newton. Fluid Mech..

[17] Sofos DF, Karakasidis TE, Liakopoulos A (2010). Effect of wall roughness on shear viscosity and diffusion in nanochannels. Int. J. Heat Mass Transf..

[18] Horn RG, Smith DT, Haller W (1989). Surface forces and viscosity of water measured between silica sheets. Chem. Phys. Lett..

[19] Kannam SK, Todd BD, Hansen JS, Davis PJ (2011). Slip flow in graphene nanochannels. J. Chem. Phys..

[20] Myers TG (2011). Why are slip lengths so large in carbon nanotubes?. Microfluid. Nanofluid..

[21] Zhang YB (2006). Flow factor of non-continuum fluids in one-dimensional contact. Ind. Lubr. Tribol..

[22] Tohidi M, Toghraie D (2017). The effect of geometrical parameters, roughness and the number of nanoparticles on the self-diffusion coefficient in Couette flow in a nanochannel by using of molecular dynamics simulation. Phys. B Condens. Matter.

[23] Toghraie D, Azimian AR (2012). Molecular dynamics simulation of annular flow boiling with the modified Lennard-Jones potential function. Heat Mass Transf..

[24] Toghraie D, Azimian AR (2010). Nanoscale Poiseuille flow and effects of modified Lennard-Jones potential function. Heat Mass Transf..

[25] Alipour P, Toghraie D, Karimipour A, Hajian M (2019). Molecular dynamics simulation of fluid flow passing through a nanochannel: Effects of geometric shape of roughnesses. J. Mol. Liq..

[26] Thomas JA, McGaughey MJH, Kuter-Arnebeck O (2010). Pressure-driven water flow through carbon nanotubes: Insights from molecular dynamics simulation. Int. J. Therm. Sci..

[27] Kamali R, Kharazimi A (2011). Molecular dynamics simulation of surface roughness effects on nanoscale flows. Int. J. Therm. Sci..

[28] Khademi M, Sahimi M (2011). Molecular dynamics simulation of pressure-driven water flow in silicon-carbide nanotubes. J. Chem. Phys..

[29] Wang L, Dumont RS, Dickson JM (2013). Nonequilibrium molecular dynamics simulation of pressure-driven water transport through modified CNT membranes. J. Chem. Phys..

[30] Murashima T, Hagita K, Kawakatsu T (2021). Viscosity overshoot in biaxial elongational flow: Coarse-grained molecular dynamics simulation of ring–linear polymer mixtures. Macromolecules.

[31] Sun Q, Zhao Y, Choi KS, Mao X (2021). Molecular dynamics simulation of liquid argon flow in a nanoscale channel. Int. J. Therm. Sci..

[32] Frentrup H, Avendaño C, Horsch M, Salih A, Müller EA (2012). Transport diffusivities of fluids in nanopores by non-equilibrium molecular dynamics simulation. Mol. Simul..

[33] Giri AK, Teixeira F, Natália M, Cordeiro DS (2018). Structure and kinetics of water in highly confined conditions: A molecular dynamics simulation study. J. Mol. Liq..

[34] Duan C, Zhou F, Jiang K, Yu T (2015). Molecular dynamics simulation of planar Poiseuille flow for polymer melts in atomically flat nanoscale channel. Int. J. Heat Mass Transf..

[35] Shahbabaei M, Kim D (2017). Molecular dynamics simulation of water transport mechanisms through nanoporous boron nitride and graphene multilayers. J. Phys. Chem. B.

[36] Zhang YB (2015). The flow factor approach model for the fluid flow in a nano channel. Int. J. Heat Mass Transf..

[37] Bhadauria R, Aluru NR (2013). A quasi-continuum hydrodynamic model for slit shaped nanochannel flow. J. Chem. Phys..

[38] Ghorbanian J, Celebi AT, Beskok A (2016). A phenomenological continuum model for force-driven nano-channel liquid flows. J. Chem. Phys..

[39] Kasiteropoulou D, Karakasidis TE, Liakopoulos A (2012). A dissipative particle dynamics study of flow in periodically grooved nanochannels. Int. J. Numer. Methods Fluids.

[40] Swift MR, Orlandini E, Osborn WR, Yeomans JM (1996). Lattice Boltzman simulations of liquid-gas and binary fluid systems. Phys. Rev. E.

[41] Naris S, Valougeorgis D (2007). Boundary-driven nonequilibrium gas flow in a grooved channel via kinetic theory. Phys. Fluids.

[42] Zhang YB (2015). A quantitative comparison between the flow factor approach model and the molecular dynamics simulation results for the flow of a confined molecularly thin fluid film. Theor. Comput. Fluid Dyn..

[43] Zhang YB (2016). The flow equation for a nanoscale fluid flow. Int. J. Heat Mass Transf..

[44] Zhang YB (2016). Calculating the maximum flowing velocity of the Poiseuille flow in a nano channel by the flow factor approach model. Int. Commun. Heat Mass Transf..

[45] Zhang YB (2016). An additional validation of the flow factor approach model. Int. J. Heat Mass Transf..

[46] Zhang YB (2018). Size effect on nanochannel flow explored by the flow factor approach model. Int. J. Heat Mass Transf..

[47] Jiang C, Ouyang J, Zhuang X, Wang L, Li W (2016). An efficient fully atomistic potential model for dense fluid methane. J. Mol. Struct..

[48] Jiang C, Wang X, Liu Q, Ke C (2020). Investigation of the relationship between nanochannel width and mass transfer characteristics for dense methane nanofluidics. Int. Commun. Heat Mass Transf..

[49] Jiang C, Ouyang J, Wang L, Liu Q, Li W (2017). Coarse graining of the fully atomic methane models to monatomic isotropic models using relative entropy minimization. J. Mol. Liq..

[50] Shell MS (2008). The relative entropy is fundamental to multiscale and inverse thermodynamic problems. J. Chem. Phys..

[51] Kamal C, Chakrabarti A, Banerjee A, Deb S (2013). Silicene beyond mono-layers—Different stacking configurations and their properties. J. Phys. Condens. Matter.

[52] Jiang C, Ouyang J, Liu Q, Li W, Zhuang X (2016). Studying the viscosity of methane fluid for different resolution levels models using Poiseuille flow in a nano-channel. Microfluid. Nanofluid..

[53] Bhadauria R, Aluru N (2013). A quasi-continuum hydrodynamic model for slit shaped nanochannel flow. J. Chem. Phys..

[54] Jiang C, Liu Q, Wang X (2019). Direct investigation of methane nanofluidic microstructure and system dynamics in rough silicon nanochannel. Microfluid. Nanofluid..

[55] Jiang C, Ouyang J, Wang L, Liu Q, Wang X (2017). Transport properties and structure of dense methane fluid in the rough nano-channels using non-equilibrium multiscale molecular dynamics simulation. Int. J. Heat Mass Transf..

[56] Zhang YB (2017). Transport in nanotube tree. Int. J. Heat Mass Transf..

[57] Zhang YB (2015). Novel nano bearings constructed by physical adsorption. Sci. Rep..

[58] Sofos F, Karakasidis T, Liakopoulos A (2009). Transport properties of liquid argon in krypton nanochannels: Anisotropy and non-homogeneity introduced by the solid walls. Int. J. Heat Mass Transf..

[59] Zhang YB (2019). Density and viscosity profiles governing nanochannel flow. Phys. A Stat. Mech. Its Appl..

[60] Zhang YB (2014). Review of hydrodynamic lubrication with interfacial slippage. J. Balkan Tribol. Assoc..

